# Acquired haemophilia A in an elderly patient: A case report of functional recovery through physiotherapy

**DOI:** 10.1016/j.ijscr.2023.108769

**Published:** 2023-09-02

**Authors:** Roberto Tedeschi

**Affiliations:** Department of Biomedical and Neuromotor Sciences, Alma Mater Studiorum, University of Bologna, Bologna, Italy

**Keywords:** Acquired haemophilia-A, Rehabilitation, Elderly patient, Physical therapy, Case report

## Abstract

**Introduction:**

Acquired haemophilia-A, although uncommon in elderly patients, poses significant clinical challenges, especially when associated with profound musculoskeletal complications. The potential mimicry of hematomas as tumors further complicates the diagnostic process.

**Case presentation:**

An 85-year-old male, with a remote history of hypertension, benign prostatic hyperplasia, and right inguinal hernia, presented with acute pain in the left lower limb, functional limitation, and deep hematomas in the ileopsoas and axillary region. Initial suspicions of a sarcomatous lesion in the ileopsoas, based on radiological findings, were refuted following histopathological examinations, which confirmed the presence of necrotic hemorrhagic tissue. The patient underwent a one-month physical therapy regimen, targeting lower extremity muscles, especially around the hip joint.

**Clinical discussion:**

The severity of the presentation and the involvement of vital muscles like the ileopsoas and quadriceps underscored the importance of comprehensive rehabilitation. Consistent therapeutic interventions, targeting muscle strength and joint function, demonstrated marked improvement as evidenced by the HJHS, HAL, and FISH scores. The multidisciplinary approach, entailing hematological, rehabilitative, and supportive measures, was paramount in ensuring holistic patient recovery.

**Conclusions:**

Acquired haemophilia-A in the elderly necessitates an integrative care approach, encompassing accurate diagnosis and tailored therapeutic interventions. This case emphasizes the transformative potential of dedicated physiotherapy in managing the musculoskeletal implications of this bleeding disorder, underscoring the value of early intervention and comprehensive care.

## Highlights

• Elderly patient with Acquired Haemophilia-A: a diagnostic challenge.
• Ileopsoas hematoma: An uncommon complication of haemophilia.
• MRI & TC imaging essential for diagnosis and treatment planning.
• Focused physiotherapy regimen aids in functional recovery.
• Rehabilitation critical for independence and quality of life in elderly.

## 1 Introduction

Haemophilia A is one of the most recognized congenital bleeding disorders, with its etiology rooted in a deficiency or dysfunction of coagulation factor VIII. Traditionally identified as a condition manifesting from birth, haemophilia A emerges through an X-linked inheritance pattern, affecting approximately 1 in 5000 male births [1]. However, there are rare instances where this disorder presents or is diagnosed in advanced age, offering a set of unique clinical challenges scarcely discussed in medical literature [2].The typical manifestations of haemophilia, including spontaneous and prolonged bleeding, become particularly concerning when they afflict elderly patients [2,3]. These bleedings often primarily target the joints but are not restricted from involving muscles, soft tissues, and other organs. The presence of other age-related comorbidities, combined with an inherently reduced recovery capability, makes the management of these patients especially intricate [4]. Despite the traditional emphasis on the hematological treatment of haemophilia A, surprisingly little has been deliberated on the physiotherapeutic approach specific to elderly patients with this condition. Physiotherapy, with its potential to foster functional recovery, alleviate pain, and enhance the quality of life, could play a pivotal role in the care of this distinct population [[5], [6], [7], [8]]. In this case report, we aim to present a detailed clinical case of an elderly patient diagnosed with acute haemophilia A and a pronounced reduction in factor VIII. Beyond examining the diagnostic and therapeutic challenges, we focus on the importance and efficacy of a targeted physiotherapeutic approach, underscoring the need for further research and discourse in this emerging field. The patient received treatment in a university-affiliated hospital renowned for its comprehensive care. Following discharge, in a domiciliary setting, the patient's physiotherapy was managed privately by a professional physiotherapist specializing in rehabilitative care for haemophilic patients.

## 2 Case presentations

An 85-year-old patient, living with his wife, was functionally independent before being diagnosed with acute acquired haemophilia A. He was complicated by deep hematomas in the right axillary cavity and the left ileopsoas muscle, both cutaneous and subcutaneous. The hematoma of the ileopsoas on the axial plane measured 10 × 7 cm at the time of hospital admission, which reduced to 9 × 5 cm at discharge. Remote medical history includes systemic arterial hypertension, benign prostatic hyperplasia, and a right inguinal hernia. Recent medical history: the patient reported lumbar pain extending to the left leg and upon recommendation from a Physical Medicine and Rehabilitation specialist, he began treatment with anti-inflammatory and intramuscular steroids, suspecting lumbar sciatica. A week later, he was hospitalized due to worsening pain symptoms associated with numbness on the anterior thigh surface and resulting functional limitations. He also reported hematomas on the left thigh and beneath the right axillary cavity. Several instrumental investigations, including lumbosacral Magnetic Resonance Imaging (MRI) and a total body Tomography Computed (TC) scan, were conducted. The CT scan of the patient revealed a well-circumscribed, non-enhancing lesion in the left ileopsoas region, consistent with a hematoma. The dimensions were suggestive of its considerable size, pressing against adjacent structures and likely contributing to the patient's pain and mobility limitations. These findings were instrumental in ruling out other differential diagnoses, particularly given the initial suspicion of a sarcomatous lesion compromising vascular and nerve structures. Additionally, a large right subfascial hematoma was identified without signs of active bleeding. Histopathological examinations were subsequently conducted with excisional biopsy, showing no neoplastic lesions but multiple fragments of necrotic hemorrhagic tissue. Further diagnostic refinement found a complete absence of factor VIII. After seven days of specific therapy (factor VIII), he required high-dose, long-term steroid therapy and was also advised to take vitamin D. During hospitalization, he underwent physiotherapy to regain ambulation. At discharge, he was using a 2-wheel walker with crutches and required caregiver assistance. He was evaluated at home the day after discharge. A general pain scale administered showed an Numeric Pain Rating Scale (NPRS) [9] 2/10 focused on the left hip joint. Objective examination revealed maintained passive mobility in all joint planes and foci. Active mobility was limited in the left limb, with flexion-extension deficits from a supine position and inability to flex the left hip against gravity with an extended knee without assistance. The strength was deficient in the area innervated by the left femoral nerve, scale Medical Research Council Muscle Strength Scale (MRC) 2/5 for the ileopsoas muscle and the femoral quadriceps, consistent with nerve entrapment due to the still-present hematoma. Administered scales revealed difficulty moving in bed, reaching and maintaining a seated position without upper limb support. Tactile sensitivity preserved, kinesthetic sensitivity preserved. Upright position maintained with significant aid and walker for hand support, assisted walking over short distances. Administered scales included Haemophilia Joint Health Score (HJHS) [10] 15/126, Haemophilia Activity List 55/100 (HAL) [11], and Functional Independence Score for Haemophilia (FISH) [12] 11/32. This case study adheres to the SCARE [13] (Surgical Case Report) guidelines for reporting surgical case studies. The SCARE guidelines aim to enhance the transparency and completeness of reporting surgical cases, providing a structured framework that facilitates accurate communication and assessment of surgical experiences.

## 3 Clinical findings

• **Subjective Reports:**
  • Lumbar pain extending to the left leg
  • Numbness on the anterior thigh surface
  • Functional limitations in mobility
  • Pain at the left hip joint (NPRS 2/10)
• **Physical Examination:**
  • Hematomas: Observed on the left thigh and beneath the right axillary cavity.
  • Mobility:
    o Passive mobility maintained in all joint planes and foci.
    o Active mobility limited in the left limb, with flexion-extension deficits from a supine position. Inability to flex the left hip against gravity with an extended knee without assistance.
  • Strength: Deficient in the area innervated by the left femoral nerve (MRC 2/5) for the ileopsoas muscle and femoral quadriceps.
  • Sensitivity: Tactile and kinesthetic sensitivity preserved.
  • Station: Upright position maintained with significant aid; requires walker for hand support and assistance for walking.
• **Instrumental Investigations:**
  • Lumbosacral MRI and Total Body TC: Suspected sarcomatous lesion in the left ileopsoas, compromising vascular and nerve structures. Voluminous right subfascial hematoma without signs of active bleeding observed.
  • Histopathological Examination: Absence of neoplastic lesions; presence of multiple fragments of necrotic hemorrhagic tissue.
  • Coagulation Factors Assay: Complete absence of factor VIII detected

The HJHS score is a tool specifically designed to assess joint health in people with haemophilia. Joint bleeds are a common complication in people with haemophilia, and over time, repeated bleeds can lead to joint damage, pain, and decreased function. The HJHS was developed to provide a standardized and objective way to assess and monitor joint health in this population.

The HAL is a self-report questionnaire developed to measure health-related functional status specifically in adult patients with haemophilia. The questionnaire takes into consideration the physical limitations and social issues that are unique to this patient group. It is an essential tool for gauging how haemophilia impacts the daily life of patients, and its outcomes are useful for both clinical and research purposes.

The FISH is a performance-based tool developed to assess functional independence in daily activities specifically for people with haemophilia. Unlike some self-reported measures, FISH evaluates actual performance in certain tasks. It provides insights into how haemophilia and its associated complications impact a person's functional ability in daily life.

### 3.1 Therapeutic intrevention

**Active Exercise Regimen:**
  1. **Tibio-tarsal Flexion and Extension** (while lying supine)
    o Duration: 4 min
    o Recovery: 1 min
  2. **Hip Flexion/Extension** (alternate movements while lying supine)
    o Duration: 4 min
    o Recovery: 1 min
  3. **Hip Flexion** (with the knee extended, while lying supine)
    o Duration: 4 min
    o Recovery: 1 min
  4. **Hip Abduction and Return** (with the knee flexed, while lying supine)
    o Duration: 4 min
    o Recovery: 1 min
  5. **Bridge Exercise** (hip lift while lying supine)
    o Duration: 4 min
    o Recovery: 1 min
  6. **Abdominals** (bringing knee to chest while lying supine)
    o Duration: 4 min
    o Recovery: 1 min
  7. **Knee Flexion and Extension** (while sitting)
    o Duration: 4 min
    o Recovery: 1 min
  8. **Sit to Stand** (repeatedly rising from and sitting down on a chair)
    o Duration: 4 min
    o Recovery: 1 min
  9. **Alternating Forward and Backward Stepping** (in an upright/orthostatic position)
    o Duration: 4 min
    o Recovery: 1 min
  10. **Walking with a 4-wheeled walker**
    o Duration: 4 min

This exercise regimen should be performed at least once daily for a span of one month [[14], [15], [16]].

## 4 Follow-up and outcomes

**After One Month of Treatment:**
• **Pain Assessment:**
  o Numerical Pain Rating Scale (NPRS) for the left hip: 0/10 - Indicating no reported pain related to the left hip.
• **Muscle Strength Assessment:**
  o Medical Research Council (MRC) scale for the left ileopsoas: Scored 3 - Indicating moderate muscle strength.
  o MRC scale for the left quadriceps: Scored 4 - Indicating good muscle strength, just below normal.
• **Functional and Activity Measures:**
  o Haemophilia Joint Health Score (HJHS): 5/124 - Demonstrating a substantial improvement in joint health and function.
  o Haemophilia Activity List (HAL): 75/100 - Indicating an improved ability to perform daily activities.
  o Functional Independence Score for Haemophilia (FISH): 22/32 - Reflecting better functional independence in daily tasks.
• **Clinical Observations:**
  o Significant reduction in all hematomas.
  o The patient can walk within a domestic environment without the support of walking aids such as a walker.

## 5 Discussion

Haemophilia-A is a renowned congenital bleeding disorder, but its acquired variant is less frequently seen, especially in the elderly [17]. The peculiarity of this case doesn't only lie in the advanced age but also the musculoskeletal complications' severity.

The deep hematomas, particularly that of the ileopsoas region measuring 10 × 7 cm at admission and reducing to 9 × 5 cm at discharge, presented a unique set of challenges. Their presence accounted for the patient's pain and limited mobility. These hematomas also introduced diagnostic dilemmas, manifested by the initial sarcomatous lesion suspicion in the ileopsoas. Hematomas in those with bleeding disorders can clinically and radiologically mimic tumors, underscoring a meticulous differential diagnosis.

Given the patient's total absence of factor VIII [18], surgical intervention, including drainage, was deemed non-advisable. The inherent risk associated with bleeding post-operation was significantly heightened. This decision gained further credence when the patient exhibited clinical improvement with high-dose corticosteroids.

Considering the bleeding risks of any surgical procedure in a hemophilic patient, physiotherapy emerged as a safer, less invasive approach compared to surgical drainage [6,19]. Before the acute Haemophilia A diagnosis, the patient was autonomous, managing other conditions like untreated prostatic/inguinal hernia pathologies that weren't the primary cause for admission.

The introduction of a tailored physiotherapy regimen, beyond basic physiotherapy, was essential. It focused not just on mobility but primarily on muscle strength enhancement, especially of the ileopsoas and quadriceps. The progress in MRC scores and the positive shifts in HJHS, HAL, and FISH scores confirmed this approach's efficacy.

A follow-up MRI is scheduled two months post-discharge to evaluate hematoma resolution further, and the findings will be pivotal for future action planning.

In conclusion, this case accentuates the need for early recognition, precise diagnosis, and comprehensive management for acquired haemophilia-A in non-traditional demographics. The symbiosis of tailored physiotherapy with medical interventions can significantly bolster quality of life and functional independence. Future research involving more extensive cohorts could solidify best practices for such challenging presentations.

## Ethical approval

Ethical approval is not a requirement at our institution for reporting individual cases or case series.

## Funding

Authors state no funding involved.

## CRediT authorship contribution statement

RT contributed to conception and design of the study; RT to data acquisition, RT to data analysis and interpretation; RT contributed to draft the manuscript; RT contributed to the critical revision for important intellectual content. All authors read and approved the final version of the manuscript.

## Consent

Written informed consent was obtained from the patient for publication of this case report and accompanying images. A copy of the written consent is available for review by the Editor-in-Chief of this journal on request.

## Guarantor

Roberto Tedeschi.

## Research registration number

None.

## Informed consent

Informed consent has been obtained from all individuals included in this study.

## Declaration of competing interest

Authors state no conflict of interest.
